# Retraction Note: Reduced immunomodulation potential of bone marrow-derived mesenchymal stem cells induced CCR4^+^CCR6^+^ Th/Treg cell subset imbalance in ankylosing spondylitis

**DOI:** 10.1186/s13075-024-03322-9

**Published:** 2024-04-19

**Authors:** Yanfeng Wu, Mingliang Ren, Rui Yang, Xinjun Liang, Yuanchen Ma, Yong Tang, Lin Huang, Jichao Ye, Keng Chen, Peng Wang, Huiyong Shen

**Affiliations:** 1 Department of Othopaedics, Sun Yat-sen Memorial Hospital, Sun Yat-sen University, 107# Yanjiangxi Road, Guangzhou, 510120 PR China; 2 Biotherapy Centre, Sun Yat-sen Memorial Hospital, Sun Yat-sen University, 107# Yanjiangxi Road, Guangzhou, 510120 PR China; 3 Department of Othopaedics, Guangdong Provincial People Hospital, 106# Zhongshan Road 2, Guangzhou, 510080 PR China

**Retraction Note: Arthritis Res Ther 13, R29 (2011)**

10.1186/ar3257

The Editors-in-Chief have retracted this article. After publication, concerns were raised regarding highly similar images in Fig. 1A-C, specifically:

- Panels A2 and C2 appear highly similar, with 180 degree rotation.
- Panels A3 and C3 appear to overlap.
- Panels B4 and C4 appear to overlap.

The authors have stated that incorrect images have been selected for this figure, and the raw data are no longer available. Due to the number of incorrect images, the Editors-in-Chief no longer have confidence in the presented data.

Huiyong Shen has stated on behalf of all authors that they agree to this retraction.

